# Revision of the *Polynemadikobraz* species-group with description of a remarkable new species from South Africa (Hymenoptera, Chalcidoidea, Mymaridae)

**DOI:** 10.3897/zookeys.783.26872

**Published:** 2018-09-03

**Authors:** Simon van Noort, Serguei V. Triapitsyn

**Affiliations:** 1 Research and Exhibitions Department, Iziko South African Museum, P.O. Box 61, Cape Town, 8000, South Africa Iziko South African Museum Cape Town South Africa; 2 Department of Biological Sciences, University of Cape Town, Private Bag, Rondebosch, 7701, South Africa University of Cape Town Rondebosch South Africa; 3 Entomology Research Museum, Department of Entomology, University of California, Riverside, California, 92521, USA University of California Riverside United States of America

**Keywords:** Africa, Afrotropical region, identification key, ovipositor, *Polynemadikobraz* species-group, taxonomy

## Abstract

A new Afrotropical species of *Polynema* Haliday, 1833 (Hymenoptera: Mymaridae), Polynema (Polynema) sagittaria van Noort & Triapitsyn, **sp. n.**, is described and illustrated based on specimens collected in the Cape Floral region in south-western South Africa. This species is morphologically closely related to the recently described Polynema (Polynema) dikobraz Triapitsyn, 2017 from Madagascar, both species possessing enlarged spine-like microtrichia on the fore wing disc that are unique among all the known world fairyflies. This new species belongs to the informal *dikobraz* species-group of the nominate subgenus of *Polynema*, which previously was only known from Madagascar. In addition, *P.sagittaria* has the ovipositor extending ventrally under the mesosoma to well in front of the head, in a bow-like curve, and housed in a narrow, anterior elongation of the metasoma, the basal sac. Occurrence and possible significance of such a bizarre ovipositor in other Mymaridae is discussed. All images and online keys are available on www.waspweb.org

## Introduction

The Afrotropical mymarid fauna is poorly known, with only 21 species of the extremely diverse and species-rich genus, *Polynema* Haliday, 1833, described from the region. Most of these are known from only two countries: Democratic Republic of the Congo, as a result of the Belgian taxonomist H. R. Debauche's (1949) description of 11 species of *Polynema*, under the name *Maidliella* Soyka, 1946, a synonym of *Polynema* (the single species he described as a *Polynema* is now in *Stephanodes* Enock, 1909), and Girault's (1917a, 1917b) description of 5 species from Tanzania. However, at least two of the species described by Debauche do not belong in *Polynema* (Triapitsyn and Aquino 2010), and the identity of Girault's species will need to be verified, based on a study of their type specimens. Three additional species are also known from Democratic Republic of the Congo, Senegal, and South Africa (Annecke and Doutt 1961, Ghesquière 1942, Risbec 1951). Triapitsyn (2017) described a remarkable new species with highly modified wing setation, Polynema (Polynema) dikobraz Triapitsyn, 2017, from Madagascar and placed it in the informal *dikobraz* species-group. This species has unique, enlarged spine-like microtrichia on the fore wing disc.

As part of a comprehensive, ongoing 26 year inventory survey of Afrotropical Hymenoptera by the senior author, four females of a continental African species belonging to the *P.dikobraz* species-group were collected in the Western Cape Province of South Africa. A fifth female from the same region was located in the CNCI collection in Ottawa, Canada. They possess the same remarkable fore wing microtrichia and also have a unique, anteriorly projecting ovipositor, the first described for *Polynema*. Because this is the first continental African species in a unique species-group of *Polynema* and the need to provide a taxon name for the extracted DNA sequence, we have undertaken a revision of this small species-group. We also discuss the ovipositor structure and place it in context of the evolution of other modes of hymenopteran ovipositor adaptation. An identification key is provided to the species of the Polynema (Polynema) dikobraz group. Online Lucid identification keys and all images are available at www.waspweb.org.

## Materials and methods

Ethanol-preserved specimens were either dried using the HMDS procedure following Heraty and Hawks (1998), or using a Critical Point Dryer (Leica EM CPD300). For slide preparation the specimens were cleared in 10% KOH solution prior to dehydration through an ethanol series, final dehydration in Euparal and dissection and mounting in Canada balsam on a glass slide under coverslips.

Images were acquired at SAMC with a Leica LAS 4.9 imaging system, comprising a Leica Z16 microscope (using either a 2× or 5× objective) with a Leica DFC450 Camera and 0.63× video objective attached. The imaging process, using an automated Z-stepper, was managed using the Leica Application Suite V 4.9 software installed on a desktop computer. Diffused lighting was achieved using a Leica LED5000 HDI dome. All images presented in this paper, as well as supplementary images, are available at www.waspweb.org

Morphological terminology follows Heraty et al. (2013), Triapitsyn (2017) and the Hymenoptera Anatomy Ontology (HAO: http://portal.hymao.org) (Seltmann et al. 2012). Measurements are given in micrometers.

Codens of depositories of specimens follow Arnett et al. (1993):

**CASC**California Academy of Sciences, San Francisco, California, USA (Curator: Brian L. Fisher)

**CNCI**Canadian National Collection of Insects, Arachnids, and Nematodes, Ottawa, Canada (Curator: Sophie Cardinal)

**SAMC** Iziko South African Museum, Cape Town, South Africa (Curator: Simon van Noort)

**UCRC** Entomology Research Museum, Department of Entomology, University of California, Riverside, California, USA (Curator: Serguei V. Triapitsyn)

## Results

### Key to species of the Polynema (Polynema) dikobraz species-group

**Table:** 

	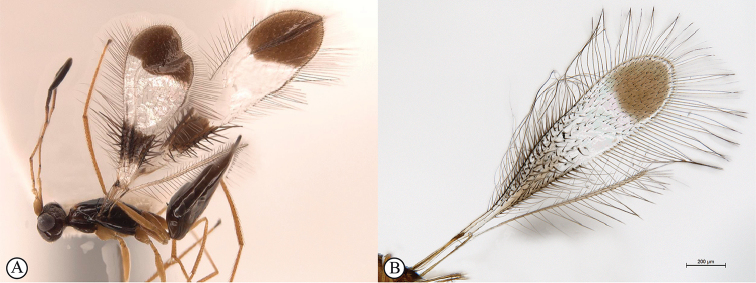	
1	Fore wing with enlarged spine-like microtrichia on basal third of disc (A, B) (Polynema (Polynema) dikobraz species-group)	**2**
	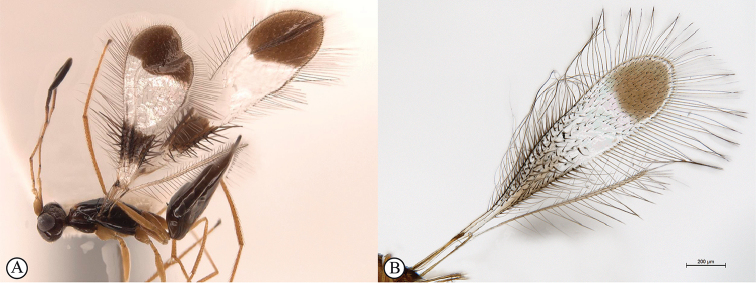	
–	Fore wing disc with microtrichia normal, evenly-sized across disc (a, b)	**all other *Polynema* species**
	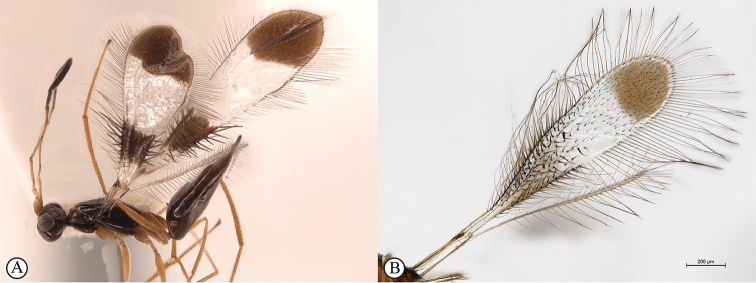	
2	Ovipositor extending forward underneath the body to in front of the head (A); modified fore wing disc microtrichia numerous and shorter (B)	***P.sagittaria* sp. n.**
	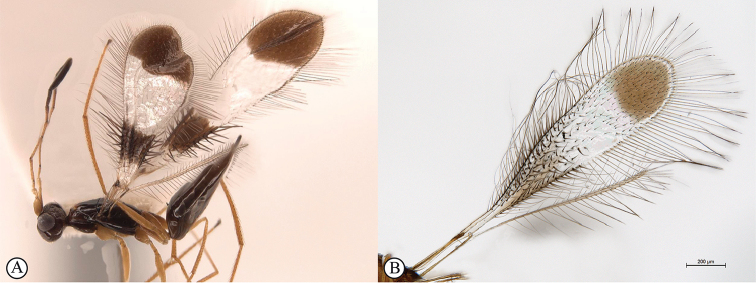	
–	Ovipositor normal, not extending forward underneath the body (a); modified fore wing disc microtrichia less numerous and longer (b)	**3**
	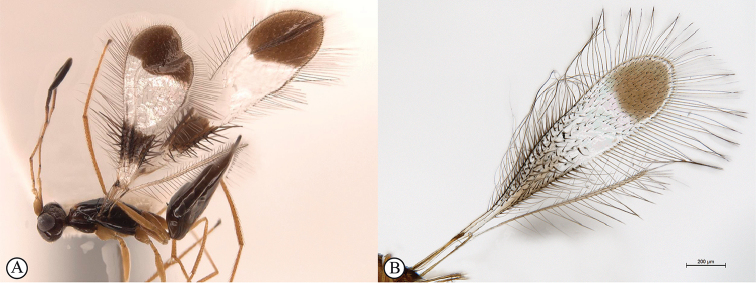	
3	Longest modified wing spine marginally longer than wing width at point of spine attachment (A); mesosoma dark brown (A); first flagellar antennal segment equivalent to pedicel length (A, B)	***P.dikobraz* Triapitsyn**
	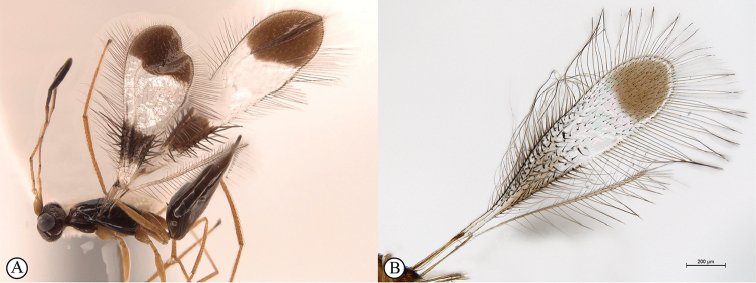	
–	Longest modified wing spine far longer than wing width at point of spine attachment (a); mesosoma mostly light brown with some dark brown areas (a); first flagellar antennal segment longer than pedicel length (a, b)	***Polynema* species near *P.dikobraz***

#### Polynema (Polynema) dikobraz

Taxon classificationAnimaliaHypocrealesClavicipitaceae

species-group

##### Diagnosis.

The *Polynemadikobraz* species-group is characterized by the possession of modified microtrichia on the fore wing disc (Triapitsyn 2017).

##### Affinities.

This species-group belongs to the nominate subgenus of *Polynema* based on absence of pits near the toruli; an “open” prosternum; a characteristic short marginal vein on the fore wing; petiole attached posteriorly to the gastral tergum; and male genitalia with digital hooks (Triapitsyn and Fidalgo 2006, Triapitsyn 2017).

##### Distribution.

Madagascar, South Africa.

##### Habitat.

Associated with montane rainforest and fynbos.

#### Polynema (Polynema) dikobraz

Taxon classificationAnimaliaHypocrealesClavicipitaceae

Triapitsyn, 2017

##### Material.

*Holotype* ♀ examined, dissected under 4 coverslips on slide and almost complete (lacking a radicle of one antenna): MADAGASCAR, Prov. D’Antanarivo 3 km 41°NE Andranomay, 11.5 km 147° SSE Anjozorobe, el. 1300 m 5–3.xii.2000, 18°28'24"S, 47°57'36"E, Fisher, Griswold et al. California Academy of Sciences Montane rainforest, MT, coll code BLF2372, CAS LOT # 005501, Mounted at UCR/ERM by V.V. Berezovskiy 2011 in Canada balsam, Polynema (Polynema) dikobraz Triapitsyn HOLOTYPE ♀, Det. by S.V. Triapitsyn 2011 (CASC).

##### Diagnosis.

Morphologically similar to the newly described species, *P.sagittaria* sp. n., both species having enlarged spine-like microtrichia. *Polynemadikobraz*, however, has a normal ovipositor, and is closely related to an undescribed species, from which it can be separated by the length of the modified wing spine and relative length of the first antennal flagellar segment.

##### Affinities.

Based on the hypothesized morphological synapomorphy of modified (long, thick) microtrichia on the fore wing disc, *P.dikobraz* is related to *P.sagittaria***sp. n.**, but has far fewer, and longer modified wing disc microtrichia. This species also has a normal ovipositor as in other members of *Polynema*, as opposed to the highly modified ovipositor of *P.sagittaria*.

##### Distribution.

Madagascar.

##### Habitat.

Montane rainforest.

#### Polynema (Polynema) sagittaria

Taxon classificationAnimaliaHypocrealesClavicipitaceae

van Noort & Triapitsyn sp. n.

http://zoobank.org/BF62E578-DB4E-4632-9AB2-A12FE7E10AF1

**Figure 1.**
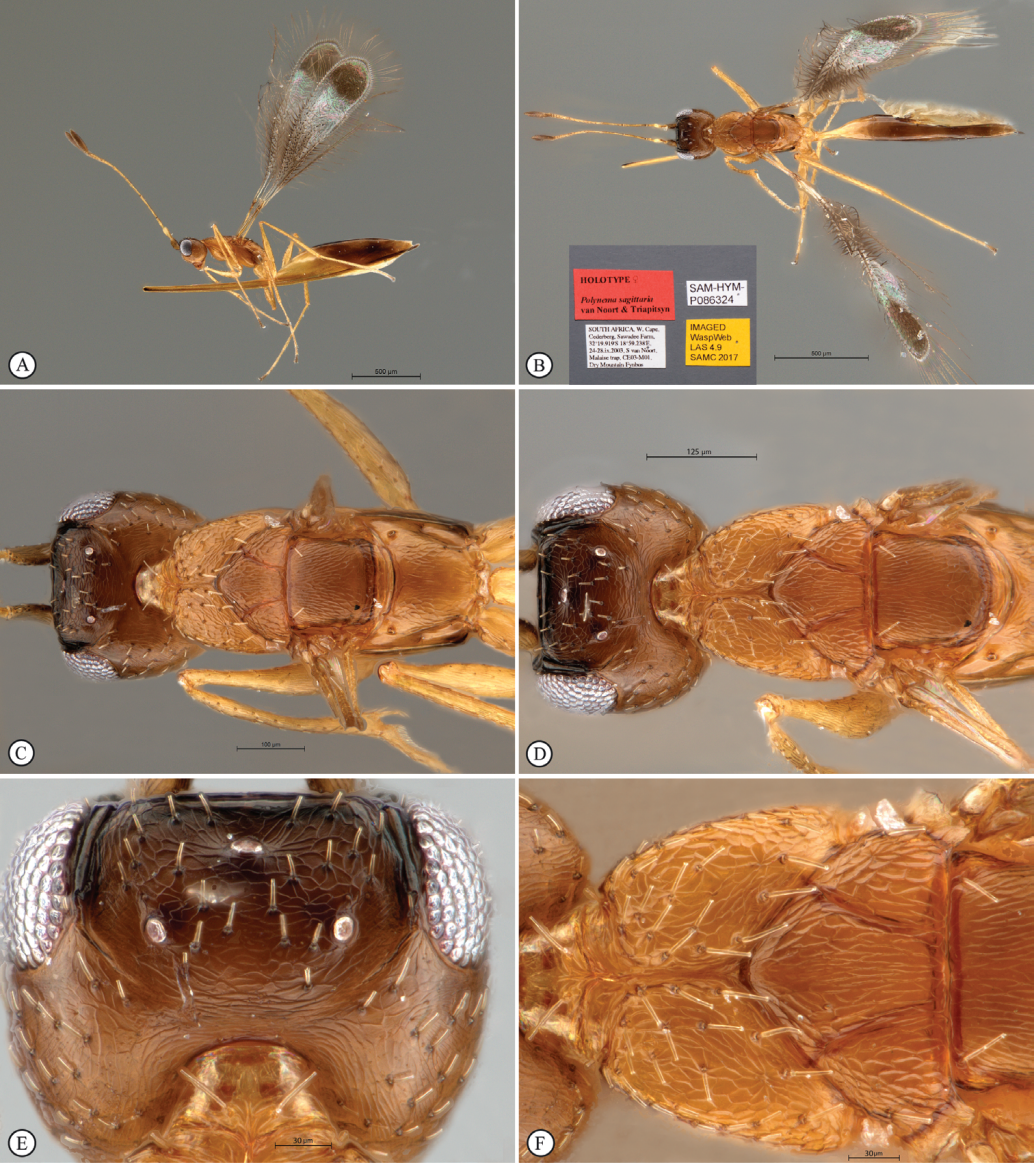
*Polynemasagittaria* Holotype female. **A** Habitus, lateral view **B** Habitus, dorsal view (inset: data labels) **C** Head and mesosoma, dorsal view **D** Head and mesosoma, anterior-dorsal view **E** Head, vertex **F** Pronotum and mesoscutum, dorsal view.

**Figure 2.**
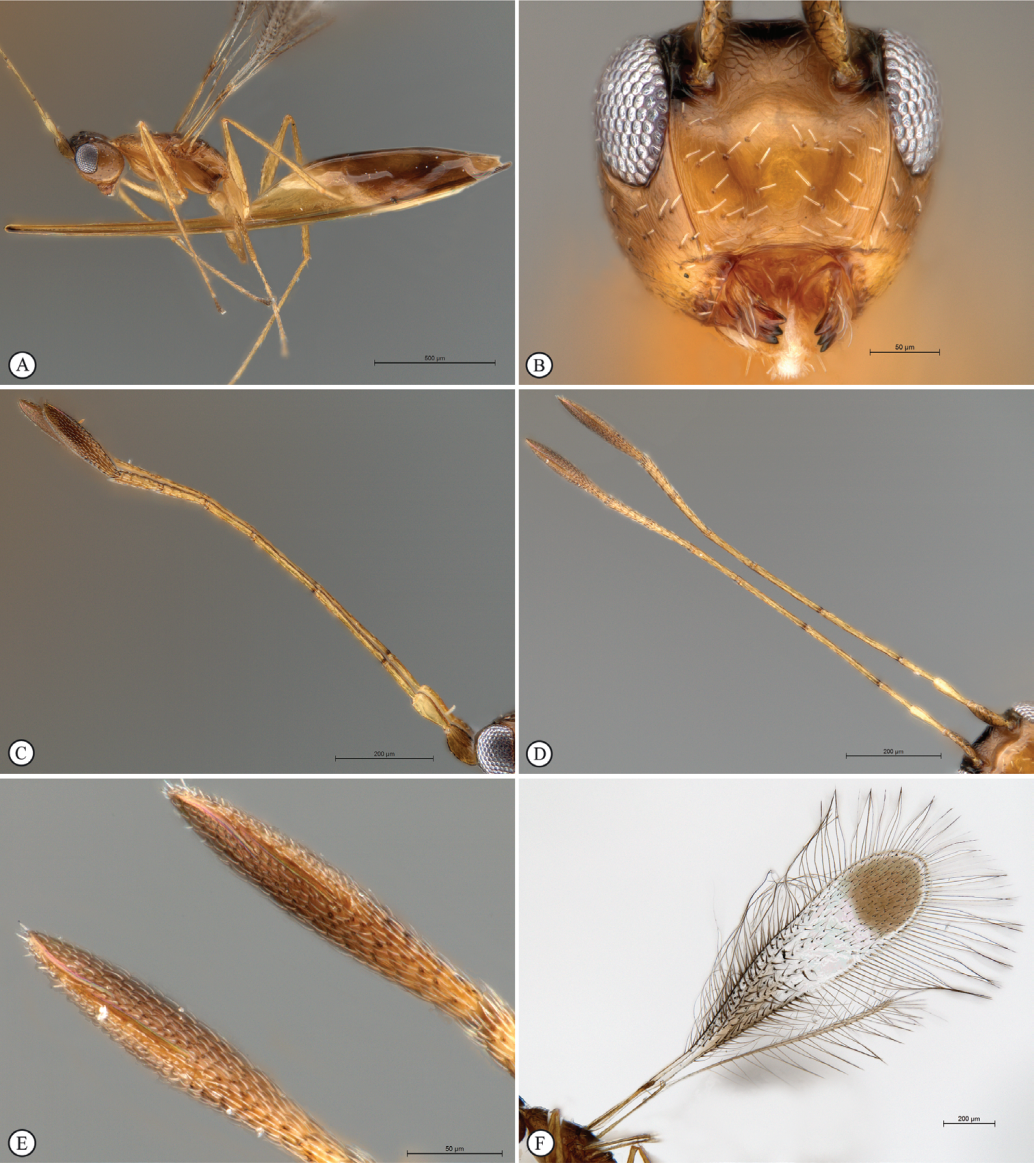
*Polynemasagittaria* Holotype female. **A** Body, lateral view **B** Head, anterior view **C** Antennae, lateral view **D** Antennae, dorsal view **E** Antennal clavae, dorsal view **F** Wings, dorsal view.

**Figure 3.**
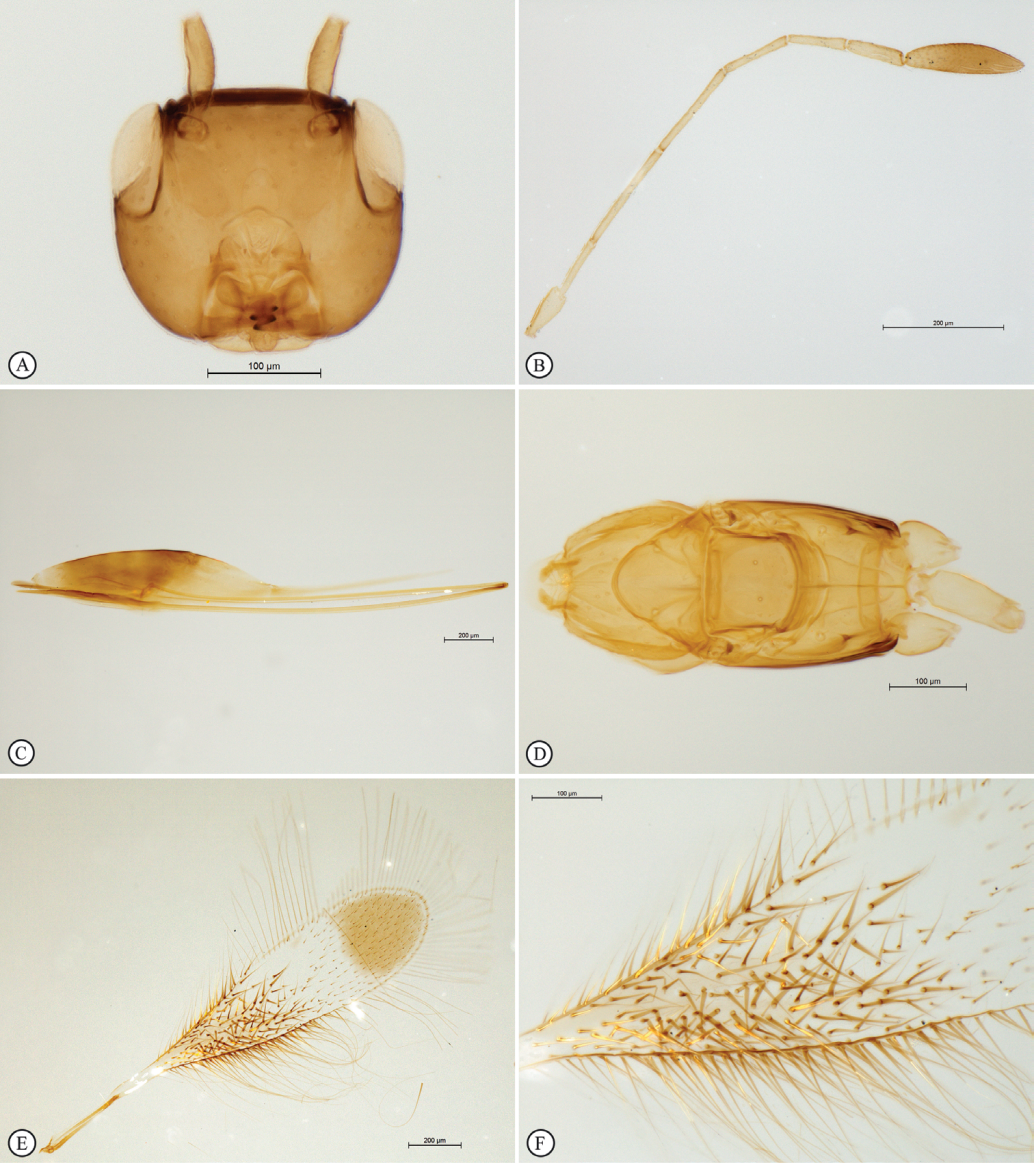
*Polynemasagittaria* Paratype female, slide-mounted. **A** Head, anterior view **B** Antenna, lateral view **C** Metasoma, lateral view **D** Mesosoma and petiole, dorsal view **E** Fore wing, dorsal view **F** Fore wing discal microtrichia, dorsal view.

**Figure 4.**
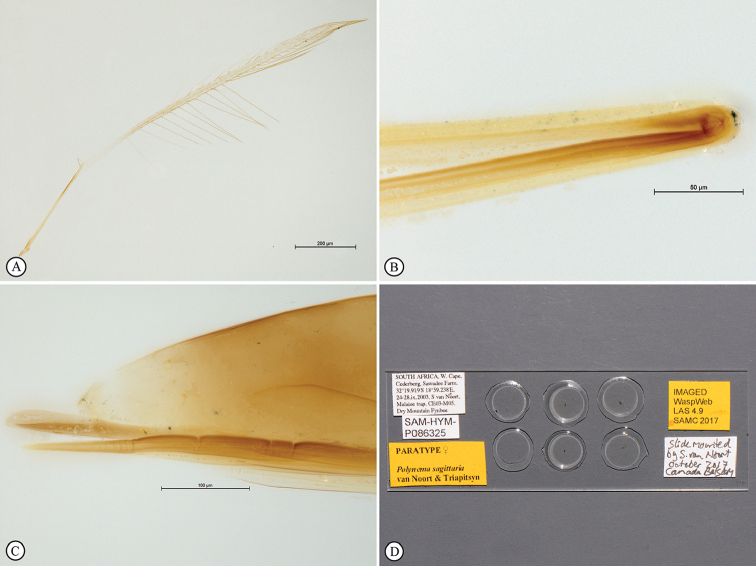
*Polynemasagittaria* Paratype female, slide-mounted. **A** Hind wing, dorsal view **B** Ovipositor distal flexion, lateral view **C** Ovipositor valves with terminal annuli and three dorsal notches on dorsal valve, sheaths and terminal metasomal segments, lateral view **D** Slide detail.

Figs 1A–F , 2A–F , 3A–F , 4A–D

##### Material.

*Holotype* ♀ (deposited in SAMC), point mounted: SOUTH AFRICA, Western Cape, Cederberg, Sawadee Farm, 32°19.92'S, 18°59.24'E, 24–28.ix.2003, S. van Noort, Malaise trap, CE03-M01, Dry Mountain Fynbos, 380 m, SAM-HYM-P086324, imaged WaspWeb, LAS 4.9, SAMC 2017. *Paratypes*. SOUTH AFRICA, Western Cape: same data as holotype, except for collecting event number: CE03-M05, and catalogue number: SAM-HYM-P086325, imaged WaspWeb, LAS 4.9, SAMC 2017 (1 ♀ on slide, SAMC); Banghoek Valley, Dwarsriviershoek Farm, 33°56.23'S, 18°57.71'E, 410 m, 22.x–27.xi.2013, S. van Noort, Malaise trap, Mesic Mountain Fynbos, BH12-FYN3-M14, SAM-HYM-P084138 (1 ♀ on slide, SAMC) [specimen lacks both hind wings; DNA was extracted using a non-destructive method, John M. Heraty's Laboratory molecular voucher D6195]; Kogelberg Nature Reserve, 34°16.48'S, 19°01.03'E, 16.x–16.xi.1999, S. van Noort, Malaise trap, K098-M44, Mesic Mountain Fynbos, last burnt c. 1988, SAM-HYM-P082695 (1 ♀ on point, SAMC); 10 km S of Citrusdal, Kornlandskloof [S32°40', E19°02], 7–9.x.1994, meadow at stand of *Herreablanda*, M. Sodelund, MT (1 ♀ on point, CNCI).

##### Etymology.

The species epithet “*sagittaria*” is Latin for armed with bow, with reference to the bowed ovipositor sheaths. Noun in apposition.

##### Diagnosis.

The highly modified ovipositor immediately distinguishes this species from all other described *Polynema* species in Africa. Morphologically similar to the recently described species, *P.dikobraz* from Madagascar, and the second undescribed Madagascan species, all three species having similar modified fore wing microtrichia.

##### Affinities.

Based on the putative morphological synapomorphy of modified microtrichia on the fore wing disc, *P.sagittaria* is clearly related to *P.dikobraz*, but has more numerous, shorter modified wing disc microtrichia than in *P.dikobraz*. The extensive external ovipositor housing is an obvious distinction within the *P.dikobraz* species-group, but this is likely to be a character state that has evolved independently in a number of mymarid genera (see discussion).

##### Distribution.

South Africa. Only known from the Western Cape Province.

##### Habitat.

Mountain fynbos, a vegetation type specific to the Cape Floral region.

##### Description of female holotype.

*Size and colour*. Total length of body, with head in prognathous position, 1700 µm. Head 205; mesosoma 511; petiole 114; metasoma 1140; ovipositor 2110 folded, 3800 total length (unfolded). Antenna: radicle 18; rest of scape 96; pedicel 91; F1 92; F2 204; F3 173; F4 115; F5 97; F6 94; clava 204. Fore wing 1670: 340; longest marginal seta 517; longest discal (spine-like) seta 117. Hind wing 1260: 23; longest marginal seta 267. Habitus (Fig. 1A, B). Head dark brown to black on vertex, face and mesosoma yellowish-brown, pedicel and anterior third of metasoma yellowish, rest of metasoma dark brown; scape dark brown, pedicel and F1–F6 yellowish, clava dark brown; legs yellowish.

*Head* (Figs 1E, 2B, 3A). Mandible 3-dentate. Antenna (Figs 2C–E): scape coriaceous, 2 × as long as wide in lateral view (excluding a short radicle); pedicel as long as F1; F2 the longest funicular segment, F3 longer than the following funicular segments, F4 a little longer than F5, the latter slightly longer than F6, F6 with one mps; clava long, 4 × as long as wide, with 4 mps (3 apical and 1 subapical).

*Mesosoma* (Figs 1C–D, 1F, 3D). Coriaceous. Pronotum mediolongitudinally divided, with numerous stout, truncate setae, collar with two strong truncate setae. Mesoscutum 1.8 × as wide as long, 0.75 × scutellar length. Axilla with 1 strong truncate seta (30 µm). Scutellum with a row of tiny, indistinct foveae on frenal line; with two anterior truncate setae. Propodeum smooth, with a truncate seta each side of the midline. Fore wing (Figure 2F) 4.9 × as long as wide; submarginal vein without seta, marginal vein with 1 dorsal seta; longest marginal seta 0.785 × maximum width of wing; disc with a distinct apical brown patch in close apposition to wing margin, occupying approximately a third of wing disc length; brownish areas in apical third of disc among spine-like microtrichia; disc setose throughout (apical two thirds of disc with normal microtrichia), with 130–140 very long, dark brown, strongly enlarged spine-like modified microtrichia (Figs 1A, 2F, 3E, 3F) on proximal brown infuscation area on wing disc. Hind wing (Figs 2F, 4A) 54.8 × as long as wide; apex of venation with a short, thickened seta; disc slightly infumate, longest marginal seta 11.6 × maximum width of wing. All coxae smooth.

*Metasoma* (Figs 2A, 3C). Petiole smooth, approximately 3 × as long as wide, slightly longer than metacoxa; ovipositor extending anteriorly in metasomal sac between the legs, 1.3 × further forward than head length; doubled back on itself in a bow-like fashion, with complex fold at anterior extremity (Figure 4B); only slightly extending beyond the metasomal apex by 0.05 × metasomal length, 1.24 × length of body in folded position (Figure 2A), 2.23 × length of body if totally extended in unfolded position; ovipositor valve with 10 closely-spaced, indistinct, terminal annuli; pre-terminally with three unevenly spaced dorsal notches (Figure 4C).

##### Variation.

Body length 1.45–1.68 mm; ovipositor (folded) length 1.93–2.00 mm in the paratype specimens.

##### Male.

Unknown.

### *Polynema* species near to *P.dikobraz*

**Material**. MADAGASCAR, Diana Region, Amber Mountain National Park, 12°31'13"S, 49°10'45"E, 1125 m, 29.i–11.ii.2001, R. Harin’Hala [1 ♀, CASC].

**Notes**. An additional species that is morphologically similar to *P.dikobraz* is known from Madagascar (Triapitsyn 2017). We refrain from describing this species here until further specimens are obtained. The single known specimen is damaged. We have, however, included this taxon in the identification key to facilitate recognition of the species.

## Discussion

For female wasps to successfully access and oviposit into hosts living within substrates, this either requires an elongate ovipositor, or an ability on the part of the female wasp to navigate through the substrate to reach the host for direct oviposition. The latter option appears to have driven evolution of various types of facial protrusions, which at least in the case of the ichneumonid genus *Genaemirum* Heinrich, 1936, has led to the hypothesis that the highly modified spade-like protrusions of the clypeus and genae are used in a shoveling manner to facilitate negotiation of the frass-filled tunnels created by the wood-boring host moth caterpillar, in order to reach the pupae for oviposition (Rousse et al. 2016). A number of species in other parasitoid wasp groups (Chalcidoidea, Diaprioidea, Figitidae, Platygastroidea, Protrotrupidae) also have facial protrusions, for which a functional hypothesis has yet to be postulated (Nielsen and Buffington 2011, Buffington and Copeland 2015, Buffington et al. in press). In terms of evolution of an elongate external ovipositor, logically it would be expected that natural selection will drive evolutionary adaptation to an optimal morphological configuration to attain a functional balance between successful host access for oviposition, and efficient flight without hindrance by a cumbersome ovipositor.

Across the Hymenoptera a number of different morphological metasomal configurations have evolved in response to this evolutionary driver of host reaching ability, with the elongate ovipositor being either housed within the metasoma in various configurations, or encased in elongated external ovipositor sheaths. Basal Hymenoptera have the ovipositor contained within the metasoma (Quicke et al. 1992, 1999, Vilhelmsen 2000, Vilhelmsen et al. 2001, Vilhelmsen and Turrisi 2011), and evolution of external ovipositor sheaths housing the valves have enabled many of the higher wasp lineages to evolve extremely long ovipositors allowing females to access hosts concealed much deeper within substrates (Quicke 2015, Vilhelmsen 2003). Life history mode, i.e., whether the strategy is one of ectoparasitism or endoparasitism, is a further underlying driver that will influence evolution of ovipositor morphology (Quicke and Fitton 1995, Belshaw et al. 2003). In concert with ovipositor elongation, evolution of the wasp “waist” has allowed for maneuvering of the metasoma to permit vertical drilling by the ovipositor (Vilhelmsen et al. 2010; Vilhelmsen 2013). For example, ichneumonoid and other wasp taxa with elongate ovipositor sheaths need to place the tip of the ovipositor at the oviposition site and to then walk backwards, which raises the metasoma and sheaths allowing for the vertical positioning of the ovipositor prior to commencement of drilling (Quicke et al. 1994, Quicke 2015). Chalcidoid wasps in a number of families, particularly the non-pollinating fig wasps have evolved a similar oviposition strategy and a number of pteromalid lineages associated with figs have evolved various adaptations of the metasomal terminal terga to lengthen the functional ovipositor (Wiebes 1966, Copland et al. 1973, Ulenberg 1985). It seems likely that *P.sagittaria* will need to employ a similar strategy of placing the ovipositor tip on the selected drilling site followed by backwards walking and simultaneous raising of the metasoma to effectively deploy the ovipositor, although mode of drilling and dis-engagement of the valves from the ovipositor sheaths will probably be different, given that the ovipositor sheaths are folded forwards underneath the body. Direct observation of ovipositing females is required to elucidate the precise mechanism, though this is a high expectation given the rarity of the species.

A number of parasitoid wasp taxa, particularly in the Platygastridae*sensu lato* (Platygastroidea), have evolved modifications of various parts of the metasoma to accommodate internal housing of the elongate ovipositor valves, in lieu of long external ovipositor sheaths (Austin and Field 1997). In a number of scelionine genera these modifications may include a telescopic ovipositor system, where the ovipositor is invaginated entirely into the body cavity, but can be extended by over three times its actual length via intersegmental membrane elongation, operated by hydrostatic pressure; or alternatively, may comprise an ovipositor system that is extended and retracted by antagonistic muscles (Austin and Field 1997, Field and Austin 1994). In various platygastrid genera, housing of the ovipositor is accommodated in an extension of the first metasomal tergite into variable forms ranging from a bump to elongate horn-like processes extending forwards above the mesosoma (Austin and Field 1997). The unusual extension of the ovipositor forwards under the body and head (in *P.sagittaria* and other mymarid genera), is a further example of evolutionary adaptation of metasomal morphology to house an elongate ovipositor. The ovipositor appears to be housed in a membranous anterior extension of a metasomal sternite. This is also evident in species of *Anaphes* Haliday, 1833 where this anterior extension of a metasomal sternite or “sac of gaster” was postulated to consist mainly of gs6 (Huber and Thuróczy 2018). Not surprisingly the configuration of the rami and associated musculature in *P.sagittaria* (Fig. 3C) diverges somewhat from the standard configuration for Mymaridae (King and Copland 1969), a structural necessity allowing for the ovipositor to initially project directly forwards instead of immediately curving ventrally in order to extend in a posterior direction. An intermediate evolutionary configuration is evident in species of *Anaphes* where the ovipositor has begun to extend forwards, housed in a broader membranous anterior extension of metasomal sternite 6 “gs6” (Huber and Thuróczy 2018), providing evidence of the evolutionary process leading to the substantial forward elongation of the ovipositor in *P.sagittaria*. The Chalcidoidea have asymmetric and overlapping halves of the upper ovipositor valve, including thickenings of the upper and lower valve walls, purported apomorphies defining the superfamily, but with exclusion of Mymaridae (Quicke et al. 1994). The upper valve of Mymaridae in cross section is symmetric and relatively simple, although its ventral wall is also thickened (Quicke et al. 1994). This sister-group relationship of Mymaridae to the remaining Chalcidoidea was supported by the molecular and morphological phylogenetic analyses of the Chalcidoidea conducted by Heraty et al. (2013). Elucidating the detailed structure and function of the ovipositor's morphological configuration in *P.sagittaria* requires further investigation using techniques such as thin-sectioning or CT scanning.

**Figure 5.**
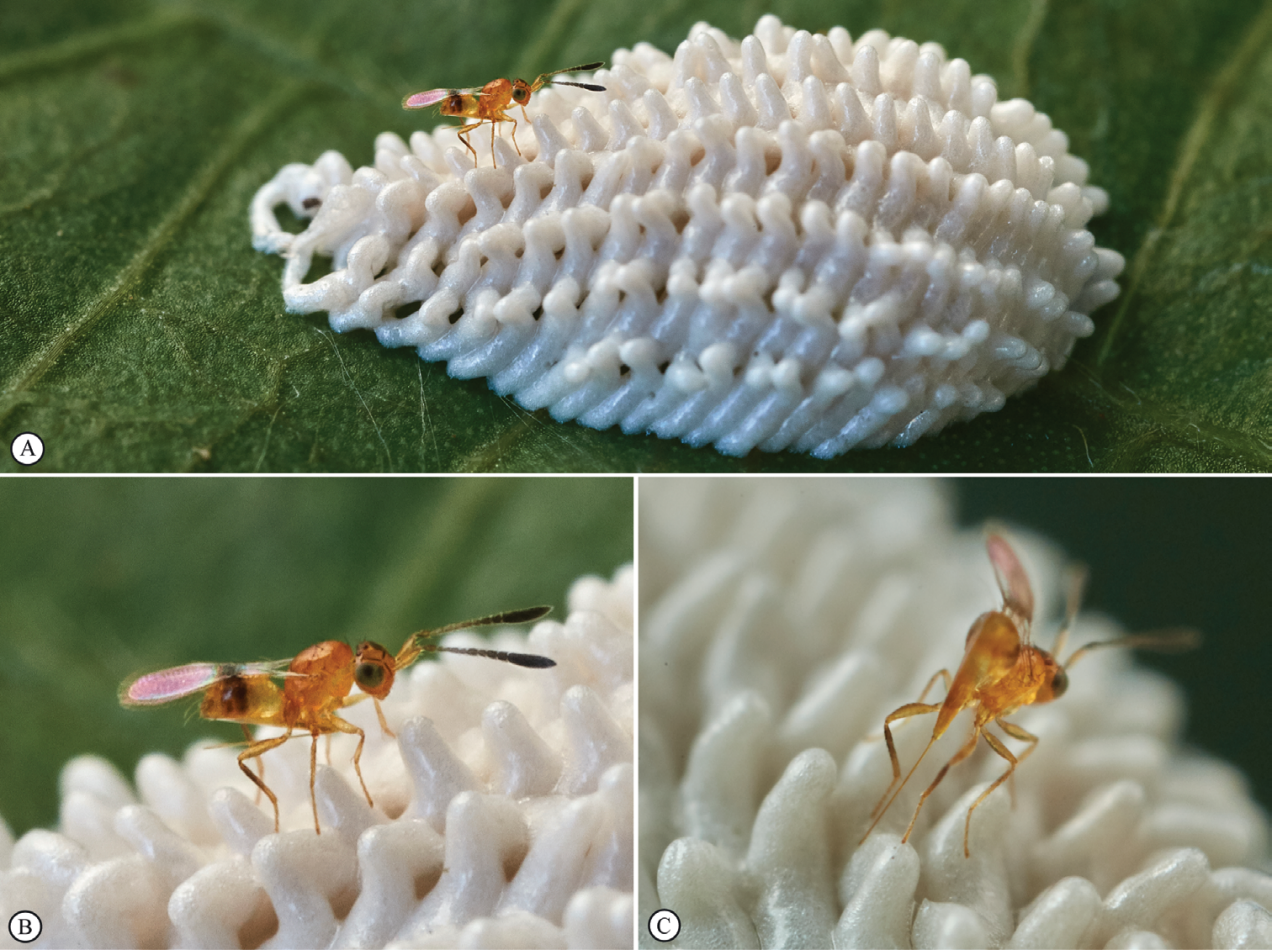
*Gastrogonatocerusmembraciphagus*. The photographs (courtesy of Paul Bertner) were taken at the Sani Lodge Prinicipal, Río Napo, Yasuní, Orellana, Ecuador, in January 2018. **A** Female (in lateral vew) on an egg mass of a treehopper (Membracidae), similar in appearance to a *Bolbonota* sp. **B** Same female (in close-up) **C** Same female (in rear view) ovipositing in that egg mass.

Among other world *Polynema* species, the ovipositor of *P.sagittaria* is unique in the extreme degree of its protrusion forward beyond its head, but a few undescribed species of *Polynema*, such as a *Polynema* species from Tanzania (one female in UCRC) and Nepal (females in CNCI), also possess such a feature, although their ovipositor is relatively shorter than in *P.sagittaria* and does not project forward beyond the head, but does reach anteriorly almost to, or even beyond the anterior margin of the mesosoma. In Mymaridae other than *Polynema*, an ovipositor similar to that in *P.sagittaria* also occurs in several other genera, such as *Gahanopsis* Ogloblin, 1946, *Gastrogonatocerus* Ogloblin, 1935, *Lymaenon* Walker, 1846 (mainly in some Australasian species), and *Neotriadomerus* Huber, 2017 (Huber 2015, 2017), and *Paranaphoidea* Girault, 1913 (Huber and Triapitsyn 2017). Thus, in Mymaridae, at least two evolutionary strategies of developing very long ovipositors can be noted. First, it is a simple lengthening of the ovipositor (and the ovipositor sheaths) beyond the posterior apex of the metasoma, with or without a large basal loop within the metasoma, but without the anterior protrusion. This happens multiple times in different, often unrelated, lineages within the family, such as in some Afrotropical species of the subgenus Anagrella Bakkendorf, 1962 of *Anagrus* Haliday, 1833 (Triapitsyn 2015), *Kalopolynema* Ogloblin, 1960 (Triapitsyn and Berezovskiy 2002), and *Omyomymar* Schauff, 1983 (Schauff 1983). The other strategy, which is found in *P.sagittaria* as well as in some members of Gonatocerini mentioned above and also in some *Australomymar* Girault, 1929 (Noyes and Valentine 1989) and *Paranaphoidea* (Huber and Triapitsyn 2017), the ovipositor strongly projects forward under the mesosoma, sometimes beyond the head. Such very long ovipositors are needed in the situations where host eggs are concealed within plant tissue, like the aerenchyma of some aquatic plants, which is the apparent case in *Kalopolynema* species having a large basal loop within the metasoma (Triapitsyn and Berezovskiy 2002), or within some other substrate or crevice. For instance, the Neotropical species *Gastrogonatocerusmembraciphagus* (Ogloblin, 1935) has a similar ovipositor to that of *P.sagittaria* although it is relatively shorter and not projecting beyond the head. Paul Bertner (personal communication) recently observed a female of *G.membraciphagus* in Ecuador examining (Fig. 5A–B) an egg mass of a treehopper (Hemiptera, Membracidae), similar in appearance to a *Bolbonota* sp. which is a known host of this fairyfly species (Triapitsyn et al. 2010), and subsequently to oviposit into the egg mass (Figure 5C). The long ovipositor is clearly seen to be used to penetrate the thick foam covering the eggs of the treehopper (Figure 5C).

In all likelihood the long ovipositor of *P.sagittaria* is a similar adaptation to reach host eggs concealed within a secondary substrate. Based on the presence of annuli at the tip of the ovipositor, which are an adaption to drill through hard surfaces (Ernst et al. 2013, Quicke et al. 1999, Le Ralec et al. 1996), we hypothesize that the host eggs themselves, or the substrate within which they are concealed is hard.

## Supplementary Material

XML Treatment for Polynema (Polynema) dikobraz

XML Treatment for Polynema (Polynema) dikobraz

XML Treatment for Polynema (Polynema) sagittaria
